# Socioeconomic and Emotional Predictors of Decision Making for Timing Motherhood Among Iranian Women in 2013

**DOI:** 10.5812/ircmj.13629

**Published:** 2014-02-05

**Authors:** Nourossadat Kariman, Masoumeh Simbar, Fazlollah Ahmadi, Abou Ali Vedadhir

**Affiliations:** 1 Department of Midwifery and Reproductive Health, Shahid Beheshti University of Medical Sciences, Tehran, IR Iran; 2 The Research Center for Safe Motherhood, Department of Midwifery and Reproductive Health, Shahid Beheshti University of Medical Sciences, Tehran, IR Iran; 3 Department of Nursing, Faculty of Medical Sciences, Tarbiat Modares University, Tehran, IR Iran; 4 Department of Antropology, Tehran University, Tehran, IR Iran

**Keywords:** Marriage, Social Support, Hope, Social Class

## Abstract

**Background:**

Decision making for timing motherhood is one of the vital aspects of reproductive health. Separating sexual relationship from having a child has led to a different and unprecedented lifestyle in human history.

**Objectives:**

The objective of this study was to determine the socioeconomic and emotional factors predicting decision making for timing motherhood among Iranian women using the statistical softwares of IBM SPSS 21 and LISREL 8.8.

**Patients and Methods:**

This cross-sectional study enrolled 820 primiparous women from different hospitals across the country using multistage random sampling method in 2013. The tools of the study were enrich marital satisfaction, socioeconomic status, perceived social support, hopefulness, and life regard index. The data was analyzed using SPSS 20 and LISREL 8.8.

**Results:**

The results revealed that among direct pathways, marital age (β = 0.62) was the most effective predictor of timing motherhood. The hopefulness had an inverse association with timing motherhood through inverse effect of marital satisfaction. Moreover, marital satisfaction (β = -0.09), perceived social support (β = -0.09), and life regard index (β = 0.01) had an inverse effect on timing motherhood. Marital satisfaction had a non-causal effect of 0.024.

**Conclusions:**

Marital age, and socioeconomic status had a direct association, and hopefulness and marital satisfaction had an indirect one with Iranian women’s decision for timing motherhood. Therefore, this is the responsibility of policy-makers and healthcare providers to advise women by providing appropriate interventions and facilities.

## 1. Background

Decision making for timing motherhood is an important aspect of the reproductive health because pregnancy at high risk ages (younger than 19 and older than 35 years) has a lot of unfavorable complications and outcomes for women, family and society. Currently, the principle of having a child and its timing is questioned by most people. Separating sexual relationship from having a child has led to a different and unprecedented lifestyle in human history (1). Increased rate of pregnancy at the two limits of fertility age has been a major health issue during the past few decades. One of the most important changes in fertility behavior is pregnancy at older ages (2). Delaying pregnancy to after 35 years is a global trend (3). In developed countries like the US, the first pregnancy at 35-39 years, and pregnancy at 40-44 years are increased to 2%, and 70%, respectively (4). The trend of advance maternal age (AMA) increased to 40% in Canada, Sweden, Denmark, Norway, Finland, Italy, and Spain (5, 6). Furthermore, about 11 million women of 15-19 years old give birth annually (11% of all pregnancies), of whom 95% occur in low to moderate income countries. Twenty-three percent of disability-adjusted life year (DALY) is related to pregnancy and childbirth in this age group (7). The objectives four and five of the Millennium development goals, indexes of international conference on population and development (ICPD) and fertility health regarding mother’s health improvement, reduced children mortality, fertility rate at 15-19, and maternal mortality rate show the importance of pregnancy at those ages and its consequences. Age specific fertility rate in Iranian adolescents has been 25 per 1000 in 2010, to 35 per 1000 in 2011, based on the WHO reported (8). On the basis of the latest census, fertility rate of adolescents (15-19 years old) in Tehran Province was reported 23.3 per 1000 (9). Furthermore, a survey of fertility in 22 municipal districts of Tehran showed a change in fertility age pattern due to delayed pregnancy. The results revealed that fertility rate in Tehran is basically due to intentional contraception by women in early and middle years of fertility (10). Pregnancy complications in older ages include abortion (11-13), gestational hypertension, gestational diabetes, cesarean section, chromosomal abnormalities (11, 14-16), stillbirth, intrauterine fetal death, prenatal mortality (17, 18), multiple pregnancy, need for fertility aids (15), and low birth weight and preterm delivery (19). Pregnancy during 15-19 years old is associated with complications including preterm delivery, low birth weight, postpartum hemorrhage (20, 21), fetal death, infant mortality, unsafe abortion, increased risk of maternal mortality, anemia, preeclampsia, postpartum depression, birth canal fistula (7, 22), placenta previa and intrauterine growth restriction (23). Furthermore, adolescent pregnancy leads to leaving school, and is an inhibitory factor in enhancing educational, economic, health and social status of women in all over the world, and has severe drawbacks on their quality of life (7, 8, 24). Findings show that no effective strategy is available in all fields regarding contraceptive programs for adolescents. For example, if an adolescent has a positive attitude toward pregnancy, increasing their knowledge about contraception methods or their accessibility to contraception cannot prevent them from pregnancy (25). In fact, different strategies should be offered to them to prevent adolescent pregnancy appropriate for their decisions and desires. Furthermore, studies show that persuasive and threatening policies have not been successful to change women’s and men’s decision-making for pregnancy in the world. The results of a qualitative study in China showed that women’s social status and their satisfaction with their status in family was the main factor for their decision making for pregnancy rather than the governmental policies (26).

Studies show that adolescent pregnancy is a complex subject and there is no clear reason for their decision (25). Meanwhile a meta-analytical research showed that the reasons for delayed pregnancy are as complex as adolescent pregnancy, and women are not aware of associated risks with such pregnancies. Healthcare providing centers do not have appropriate information and care for informed decision making about the age and the decision making process of pregnancy, and such services are not provided for them (27), while women are the final decision makers for pregnancy (3). Most women are exposed to pregnancy and have no choice but making decisions about pregnancy, contraception, and decision about the best timing motherhood. There is little knowledge about decision making for pregnancy, timing motherhood, women’s and men’s attitude toward decision making for pregnancy and their strategies for making better decisions (28). The role of socioeconomic factors in decision making has not been considered completely. Creating a link between socioeconomic determinants and micro-variables of fertility has still remained a challenge in population-based researches (29). In the first phase of this research, a qualitative study was conducted to determine the concepts and factors influencing decision making for timing motherhood including marital status, hopefulness, social support and women’s attitude to ward life. Although there are standard tools to determine and measure those concepts, the quota and position of these concepts in decision making for timing motherhood were assessed using this tool in the second phase of the study. There is no accurate information about the causing agents of timing motherhood among Iranian women, and that appropriate interventions by authorities and healthcare providers need designing and implementing researches in this regard, hence this study was conducted on the socioeconomic and emotional predictors of timing motherhood in Iranian women in 2013. It is expected that the authorities of Iranian healthcare system will benefit from the results of this study and receive precise information about the causing agents and appropriate interventions. Furthermore, other researchers can design interventional studies based on the findings of this research.

## 2. Objectives

This study aimed to determine sociocultural, economic and emotional predictors of timing motherhood in Iranian women in 2013 using statistical causal modeling including the Path Analysis.

## 3. Patients and Methods

This cross-sectional study was conducted on 820 pregnant women from January to May 2013. The statistical population of the study included all pregnant women admitted to selected hospitals in Iran who met the inclusion criteria. To determine the sampling centers, first, all provinces were divided into three stratums of low (< 1.70), average (1.7-2.0), and high (> 2.0) fertility rates according to the total fertility rate of 2006 (30). Then, three provinces from each stratum were randomly selected, and two cities were randomly selected and in each province, and the largest hospital was determined. With reference to the fertility rate of the Iranian provinces, the selected provinces included the following ones: Tehran, Guilan and Semnan (> 1.70), Ardebil, Fars, Kurdistan (1.70-2.0), Sistan and Baluchistan, Hormozgan and Lorestan (> 2.0). Then, hospitals with the most referrals of pregnant women were selected in each provincial center, and women going to the prenatal clinics were investigated. A quota was determined and purposive sampling was performed in each center based on the number of admitted pregnant women. To determine the sample size, the literature review and research variables were studied, and 10-15 samples per variable were considered appropriate. In this study, 30 variables were studied. The main concepts of the study were assessed by 80 items, and 3-10 samples were considered for each item (31), so 880 women were estimated for sample size by considering the dropout rate of 10%. Twenty-five women did not fulfill the inclusion criteria. Seven women were not willing to participate, and twenty-eight women were excluded due to unwanted pregnancy.

This study was approved in the 130st meeting of the Ethics Committee of the Deputy for Research of Shahid Beheshti University of Medical Sciences (May 12, 2012). Sampling began after obtaining the necessary permissions from authorities of the university and selected centers, and training the research team. Qualified women were familiarized with the objectives and methods of the research and if willing, they signed the informed written consent form. They were also reassured of the information confidentiality. They were informed that they could withdraw at any time, and their privacy was respected by researchers.

### 3.1. Participants

The inclusion criteria were being Iranian, married for the first time, and primiparous. Women with unwanted pregnancies were excluded.

### 3.2. Questionnaire Measures

The data was collected using the following questionnaires: demographics and obstetrics, factors related to decision making for pregnancy among Iranian women (design and psychometrics properties of this questionnaire was performed in the first phase of the study [the qualitative research] using grounded theory method), Enrich Marital Satisfaction Scale, Perceived Social Support, Snyder Hope Scale and Life Regard Index. The questionnaires had 80 items in two sections. Each section took about 30 min to be completed by pregnant women. For illiterate subjects, the items were read out by the researcher and the response by the subjects was marked in the questionnaire.

### 3.3. Demographics and Obstetrics Questionnaire

The questionnaire included variables as follows: age, marital age, marriage duration, ethnicity, spouse’s age, participant’s and her husband’s education, participant’s and her husband’s occupation, income, housing status, residence area per capita, family size, leisure and facilities, and obstetrics issues including gestational age, abortion, contraception method, and access to contraception.

### 3.4. Enrich Marital Satisfaction

This questionnaire was used as a reliable tool for clinical and research settings. It was applied by Fowers and Olsen (1989) in a randomized sampling on 5039 couples, and could discern satisfied from dissatisfied couples with the accuracy of 85-95% (32). The validity and reliability of this tool was assessed by Olson on 25501 married couples with 4 subscales and 35 items to determine marital satisfaction, communication, and conflict resolution. Alpha coefficient for subscale of marital satisfaction, communication, and conflict resolution were 0.86, 0.80, 0.84, and 0.83, respectively (32). The questionnaire used 5-point Likert scale, namely, the style answers of “absolutely agree”, "agree", "neither agree nor disagree", "disagree", and "totally disagree"; each item scored from 1 to 5. The questionnaire had four separate scores, and the total score of each subscale was calculated and the total raw scores were converted to percentages. This tool was translated and modified based on the Iranian context. Asoudeh found the alpha coefficient for marital satisfaction, communication, conflict resolution, and idealistic distortion scale as 0.62, 0.78, 0.78 and 0.77, respectively (33).

### 3.5. Snyder Hope Scale

Based on the Snyder’s hope theory, this scale evaluates an individual’s hope as a relatively stable personality characteristic. It was designed by Snyder as a 12-item scale in 1991 for people older than 15, and has two subscales of pathway and motivation. Answers ranged from one as "totally wrong" to four as "totally right". Items 3, 5, 7, and 11 are not scored. Hopefulness score is the sum of these two subscales. Hence, the total score ranges from 8 to 32 (34, 35). Snyder et al. reported its reliability through test-retest after 3 weeks as 0.85 for the whole scale, 0.81 for agency thinking, and 0.74 for pathways (34). In an Iranian student population, its reliability was assessed and reported as alpha coefficient of 0.82, 0.79, and 0.88 for whole scale, agency thinking and pathways (36). Kermani et al. (2011) also reported Cronbach’s alpha of 0.86, 0.77 and 0.79 for the whole scale, agency thinking and pathways. Hopefulness scores correlate with Scheier and Carver Optimism Scale to 50-60 percent. Furthermore, it negatively correlates with Beck Depression Inventory according to clinical specialists. This scale has been validated for content validity (37). Socioeconomic status was assessed by using a researcher-made questionnaire including variables of education of participant and her husband, cost per square meter of land, residence area per capita, leisure and facilities (having a private car and a computer). The correlation of these factors with the whole score was measured as 0.87. The factor analysis of the standardized total score was calculated using summary index for all participants, and its agreement with the common score of the summary index was assessed with Kappa test. Potential maximum score for socioeconomic summary index was 48 (38).

### 3.6. Perceived Social Support

The MSPSS (multidimensional scale of perceived social support) has 12 items and measures perceived support from three domains of family, friends, and significant others (39, 40). Participants completing the MSPSS should express their agreement with items on a 7-point Likert scale ranging from "strongly disagree" to "strongly agree". The score ranged from 12 to 84, where 12-48 shows low social support, 49-68 shows average social support, and 69-84 shows high social support (41). A number of studies have provided adequate psychometric properties for the MSPSS in young adults in the United States and in Europe (39, 41-43). Canty-Mitchelland Zimet (2000) found the internal reliability of 0.93 for the total score and 0.91, 0.89, and 0.91 for the family, friends, and significant others subscales on a sample of urban adolescents in the United States (41).

### 3.7. Life Regard Index

**Figure 1.**
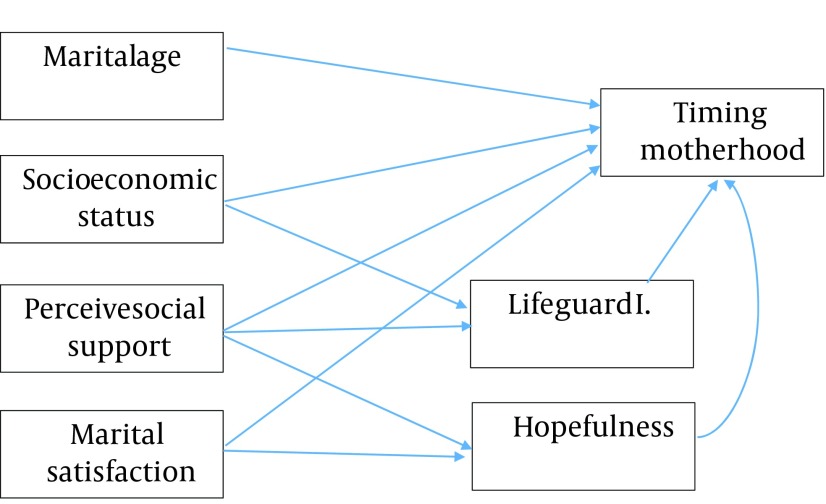
Theoretical Path Model for Social and Emotional Factors Predicting Decision Making for Timing Motherhood

Life Regard Index was designed by Batista and Almond (1973) based on positive attitude toward life. This scale was designed to evaluate seeking meaning in life (44). In 1998, this index was revised by Debats (1990) to handle its methodological problems. It has two subscales of framework, measuring person’s perception of structure and philosophy of life and their attitude toward purpose in life, and fulfillment measuring their perception of life based on their selected framework. Each scale has 14 items, of which 7 are expressed in a positive manner and 7 in a negative manner. Total scores of positive and negative items show the total score of the index. The questionnaire has three options of "agree", “have no idea”, and "disagree" with 1-3 scores, respectively. Negative items were scored inversely, and total score ranged from 14 to 42 (45). Batista and Almond reported the test-retest reliability of 0.94 for this tool. A number of studies have reported the internal consistency of this tool as Cronbach’s alpha of 0.75 to 0.87 (46). This tool was translated and used in Persian by Nasiri and Jokar’s study (2008). Its validity was assessed using factor analysis with varimax rotation (47). The reliability of the tools used in this study was assessed using internal correlation and Cronbach’s alpha coefficients for the questionnaire of Enrich Marital Satisfaction, Multidimensional Scale of Perceived Social Support, Snyder Hope Scale, and Life Regard Index as 0.84, 0.76, 0.85, and 0.78 respectively. In this study, fitness of the conceptual model was examined in order to determine the concurrent association of age at marriage, socioeconomic status, perceived social support, marital status, and hopefulness with timing motherhood (Figure 1). The data was analyzed IBM SPSS 21 and LISREL 8.8 through the path model.

## 4. Results

The mean age of 820 women was 25.21 ± 5.13 years, and their mean duration of marriage was 3.56 ± 4.75. Regarding their education, 47.3% (388 people) of the women and 45.7% (388 people) of their husbands had middle schools education. 87.3% (716 people) were housewives and 46% (377 people) of their husbands were government employees. 68.2% (559 people) of the women were born in cities, 82.3% (675 people) lived in cities and 52% (426 people) rented their residence. Most participants’ ethnicity was Fars (26.6%) and a least were Arab (8%). 32.1% (263 people) used withdrawal for contraception and all women were primiparous. The mean favorable number of children for women was reported as 2.42 ± 1.06. The statistics of mean, standard deviation (SD), maximum and minimum of main variables are presented in Table 1. 

**Table 1.** Distribution of Factors Scores

Variable	Mean ± SD	Minimum	Maximum
**Timing motherhood, y**	25.66 ± 5.76	13.00	43.00
**Marital age, y**	22.09 ± 6.64	10.00	39.00
**Hopefulness**	20.33 ± 4.32	9.00	32.00
**Marital satisfaction**	37.08 ± 4.64	20.00	48.00
**Perceive social support**	61.01 ± 12.10	23.00	84.00
**Life regard index**	31.66 ± 4.73	15.00	42.00

First, the normal distribution (by Kolmogorov Smirnov test), homoscedasticity and liner relationship were checked to perform the pathway analysis. The correlation among variables was measured using the bivariate analysis (Table 2). As shown in the Table, timing motherhood was inversely significantly correlated with hopefulness, marital satisfaction and perceived social support. Meanwhile, there was a direct significant association between marital age and socioeconomic status. Life Regard Index was directly related with timing motherhood, but the association was not significant.

**Table 2.** Correlations Among Timing Motherhood, Marital age, Hopefulness, Socioeconomic Status, Perceived Social Support, Marital Satisfaction and Life Regard Index

	Timing Motherhood	Marital Age	Hopefulness	Socioeconomic Status	Perceived Social Support	Marital Satisfaction	Life Regard Index
**Timing motherhood**	1	-	-	-	-	-	-
**Marital age**	0.713 ^a^	1	-	-	-	-	-
**Hopefulness **	-0.206 ^a^	-0.11 ^a^	1	-	-	-	-
**Socioeconomic status**	0.411 ^a^	.321 ^a^	-0.138 ^a^	1	-	-	-
**Perceived social support**	-0.23 ^a^	-0.17 ^a^	0.028	-0.113 ^a^	1	-	-
**Marital satisfaction**	-0.333 ^a^	-0.208 ^a^	0.195 ^a^	-0.353 ^a^	0.152 ^a^	1	-
**Life regard index**	0.028	0.05	-0.032	0.009 ^a^	-0.006	-0.026	1

^a^ Correlation is significant at the 0.01 level.

The goodness of fit for the research conceptual was measured using path analysis (Figure 1). Fitness indices showed that the conceptual model of the study had a good fitness, and the hypothesis of causal association of socioeconomic status, emotional characteristics and perceived social support with women’s decision making for timing motherhood was approved. Given the root mean square error of approximation (0.03) < 0.1, normal χ^2^ < 3 (1.93) and indices of GFI (goodness of fit index), CFI (comparative fit index), NFI (normed fit index) and IFI (incremental fit index) between 0.99-1 (31) showed high fitness of the model and that the associations between variables were logical according to theoretical framework of the study (Table 3). 

**Table 3.** Goodness of Fit indices for the Model ^a^

Model Index	χ^2^	df	RMSEA	GFI	NFI	CFI	IFI
	7.74	4	0.034	1.00	0.99	1	1

^a^ Abbreviations: RMSEA, root mean square error of approximation; GFI, goodness of fit index; NFI, normed fit index; CFI, comparative fit index; IFI= incremental fit index.

**Figure 2.**
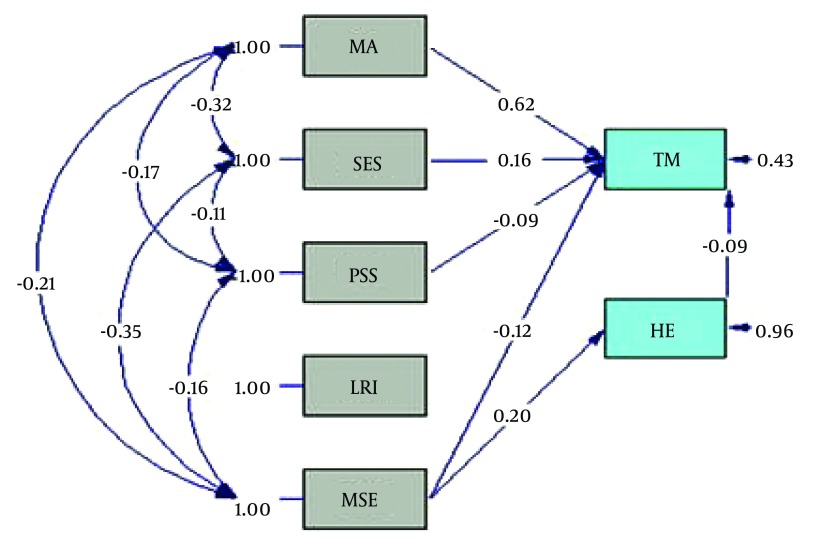
Full Empirical Model (Empirical Path Model for Effects of Marital Age, Hopefulness, Socioeconomic status, Perceived Social Support, Marital Satisfaction, and Life Regard Index on Timing Motherhood) TM, timing motherhood; MA= marital age; HE= hopefulness Snyder; SES= socioeconomic status; PSS, perceived social support; MSE= marital satisfaction enrich; LRI= life regard index.

The effect of socioeconomic, hopefulness, marital satisfaction, perceived social support, and life regard index variables on timing motherhood was studied (Figure 2). According to the diagram, marital age among the direct pathways (β = 0.62) had the highest effect on timing motherhood. Marital satisfaction had an indirect association with timing motherhood through the inverse effect hopefulness. In addition, hopefulness (β = -0.09), perceived social support (β = -0.09) and life regard index (β = -0.01) had inverse effect on timing motherhood. Hopefulness had non-causal effect of 0.024 (Table 4). The amount of factorial load of life regard index was measured as < 1.96 using T value test, and due to its insignificance at 0.05, its association with the dependent variable of timing motherhood was removed from the model.

**Table 4.** Path Coefficients for Prediction Variables of Timing Motherhood

Predictor Variables	Effect			t value	Model Coefficient
	Total	Indirect	Direct		
**Marital age**	0.62	-	0.62	0.615	25.24 ^a^
**Socioeconomic status**	0.15	-	0.15	0.147	-3.93 ^a^
**Perceive social support**	-0.09	-	-0.09	-0.088	-3.80 ^a^
**Hopefulness**	-0.09	-	-0.114	-0.091	-4.87 ^a^
**Marital satisfaction**	-0.12	-0/018	0.0138	-0.122	5.84 ^a^
**Life regard index**	-0.01	-	-0.01	-0.011	-0.49
**Marital age**	0.62	-	0.62	0.615	25.24 ^a^

^a^ Significant at the 0.05 level.

## 5. Discussion

The Path Analysis showed that marital age and socioeconomic status had a direct effect on decision making for timing motherhood in Iranian women. By increasing the marital age, education level of husband and wife, income, improved education and facilities would increase the timing motherhood. Increase in marital age and desire to remain single are increased in urban and rural areas of Iran. Increased marital age especially in rural areas is significant and could be attributed to young men’s emigration from villages and their lack of desire to return to villages or getting married with rustic villagers. Regarding the ratio of married women in 20-30 age group to whole women in that age group, it is shown that this ratio was decreased from 74.1% in 1992 to 54.8% in 2010 (48, 49). Benzis (2006, 2008) showed that age, women’s independence and financial stability are determining factors for timing motherhood (28, 50). Bayrampour and Heaman studied the demographics and obstetrics characteristics of primiparous women in both groups of older (> 35 years old), and younger (< 35 years old) women in Canada. The results showed that older primiparous women had higher education and income and were mostly employed (6). Another important factor on women’s decision making for timing motherhood is the expenses of raising children. An increase in total life expenses, besides changes in parents’ orientation in increasing the quality instead of quantity of children, decreased family desire to have children. Furthermore, increased family expenses for children’s quality has inversely affected women’s timing motherhood and number of children. The present study showed that timing motherhood, contrary to the common expectation, was not affected by women’s occupation. Latest studies and census show that women’s participation in the market is not significantly increased, and has fluctuated between 10% and 15% (51). Obtained results from experimental studies show that increased number of working hours and increased years of studying are considered increased cost- opportunity of increased number of children- and have therefore delayed the first pregnancy and low fertility rate significantly. Women’s increased education level and occupation has delayed their age at first marriage and improved their status in family and society, and all of which decrease fertility. Although equal opportunities to men have been given to women for education and working, this has not happened in family. Higher levels of education made women more powerful for decision making for housework and fertility, because such high education allows them to question their traditional roles (29). Cooke et al. studied women’s experiences, views and attitudes toward delayed motherhood through a phenomenological qualitative study. In this study, the main reasons for delayed motherhood were lack of choice for starting family, financial stability, stable relationship, health and fertility, and lack of decision making (3). The present study showed that marital satisfaction, social support and hopefulness inversely affected timing motherhood. Pathway analysis showed that better social support causes decision to have children at lower ages. In addition, the results showed that marital satisfaction (communication, conflict resolution, sexual relationship, marital cohesion and financial management) reduces timing motherhood. Hopefulness is a thought process with two parts of agency thinking and purposeful plans (fulfillment). Agency thinking is the motivational part of hope and shows person’s perception of the ability to achieve their goals (37). The study showed that hopeful women become pregnant earlier. Brayant et al. conducted a qualitative study on the role of race, ethnicity, social status, women’s perceived social status as compared with others on women’s decision for timing motherhood in the US the results showed that one-third of women had unplanned pregnancies and most of them believed in destiny in their pregnancy and had low social status in their own opinion. Women’s age, race, ethnicity, having children, and mental social status had a meaningful association with decision making for pregnancy (52). Zhenzhen studies showed that women who are satisfied with their status in family and society more often believe that they can make decision to have children alone or together with their husbands (26). Systematic reviews on delayed timing motherhood showed that independence, motivation to have a family, stable relationships, spouse’s preparedness to have children, social acceptance of delayed pregnancy age, and divorce are among effective factors on this decision (50).

People’s decision is affected by significant others, feelings, socio-cultural grounds, their interests and values. People’s values, interests and feelings are formed through confronting with different environments like family, media, education, peers and in a word, their world life. Furthermore, fertility decisions like pregnancy or contraception are influenced by social grounds and social networks of people (53). We limited the study population to the first pregnancy, because it seems that making decision for the first child is different from that of the second or other children. Although young parent’s and older parent’s decision for other children might be different from the past, it is expected that such differences are more in the decision for the first child. Decision making for timing motherhood and the number of children is a joint decision between couples. The target group in this study was women because we considered them more informed and the final decision maker in this process. In this study, only women admitted to governmental centers were studied. The results of this study cannot be generalized to women admitted to private centers and those who do not receive any prenatal care. In addition, multistage random sampling was based on general fertility, which reduces the generalizability of the results. This study is the first of its kind on the role of affective/psychological factors in decision making for timing motherhood based on concepts of a qualitative study and using a standard tool to study these concepts. The researchers recommend to conduct further studies to determine predicting factors in decision making for timing motherhood in men and women.
